# Prion Variants of Yeast are Numerous, Mutable, and Segregate on Growth, Affecting Prion Pathogenesis, Transmission Barriers, and Sensitivity to Anti-Prion Systems

**DOI:** 10.3390/v11030238

**Published:** 2019-03-09

**Authors:** Reed B. Wickner, Moonil Son, Herman K. Edskes

**Affiliations:** Laboratory of Biochemistry and Genetics, National Institute of Diabetes and Digestive and Kidney Diseases, National Institutes of Health, Bethesda, MD 20892-0830, USA; moonil.son@nih.gov (M.S.); hermane@niddk.nih.gov (H.K.E.)

**Keywords:** anti-prion systems, Sup35p, Ure2p, [PSI+], [URE3], in-register parallel beta sheet prions, Btn2, Hsp104, inositol polyphosphates

## Abstract

The known amyloid-based prions of *Saccharomyces cerevisiae* each have multiple heritable forms, called “prion variants” or “prion strains”. These variants, all based on the same prion protein sequence, differ in their biological properties and their detailed amyloid structures, although each of the few examined to date have an in-register parallel folded β sheet architecture. Here, we review the range of biological properties of yeast prion variants, factors affecting their generation and propagation, the interaction of prion variants with each other, the mutability of prions, and their segregation during mitotic growth. After early differentiation between strong and weak stable and unstable variants, the parameters distinguishing the variants has dramatically increased, only occasionally correlating with the strong/weak paradigm. A sensitivity to inter- and intraspecies barriers, anti-prion systems, and chaperone deficiencies or excesses and other factors all have dramatic selective effects on prion variants. Recent studies of anti-prion systems, which cure prions in wild strains, have revealed an enormous array of new variants, normally eliminated as they arise and so not previously studied. This work suggests that defects in the anti-prion systems, analogous to immune deficiencies, may be at the root of some human amyloidoses.

## 1. Introduction

“Prion” means “infectious protein” without the need for an accompanying nucleic acid to transmit the infection [1,2,3]. This term originated with the studies of the mammalian transmissible spongiform encephalopathies based on self-propagating altered forms of the PrP protein (includes scrapie of sheep and Creutzfeldt–Jakob disease and Kuru of humans; reviewed in Reference [4]). Most known prions are self-propagating amyloid (a filamentous β-sheet rich polymer) forms of normally soluble proteins, although there is one non-amyloid prion, namely, the [BETA] prion of yeast, which is the active form of vacuolar protease B [5]. [URE3] [6] is an amyloid-based prion of Ure2p [3,7,8,9,10], whose normal function is the regulation of nitrogen catabolism [11]. When Ure2p is largely converted to amyloid, genes encoding enzymes and transporters needed for using poor nitrogen sources are derepressed in spite of the presence of a good nitrogen source. [PSI+] [12] is an amyloid prion of Sup35p [3,13,14,15,16,17,18,19], a subunit of the translation termination factor [20,21]. When most of the Sup35p is confined to the [PSI+] amyloid, there are more frequent read-throughs of nonsense codons. [PIN+] [22] is a prion of Rnq1p [23], whose normal function is unknown [24] but which makes its presence known by (rarely) priming the formation of the [PSI+] prion. [Het-s], an amyloid-based functional prion of the HET-s protein, is part of a self-recognition system in the filamentous fungus *Podospora anserina* [25]. [Het-s] is of great interest both because of its properties [26,27] and because it differs in revealing ways from yeast prions.

## 2. Prion Variants/Strains

As has long been known for mammalian prions, yeast prion proteins with a single sequence can be the basis for a wide array of heritable, clearly distinct prions [28,29,30], called “prion variants” or “prion strains”. Variants of the yeast prions [PSI+] and [URE3] were first distinguished as “strong” vs. “weak”, meaning the strength of the prion phenotype, reflecting the degree of deficiency of the normal form of the protein. Prion variants also differ dramatically in their stability, the frequency with which they are lost on mitotic growth. There is some correlation of a variant being strong and stable or being weak and unstable, explained by the observed shorter filament size of strong filaments and longer length of weak filaments. The higher number of filament ends are believed to favor the capture of a higher proportion of the monomers and thus a “strong” phenotype [19,31,32]. Similarly, the larger number of filaments in a strong variant make the failure of a daughter cell less likely to receive no filaments and thus allows for it to be cured. However, strong unstable and weak stable variants of the [URE3] prion have been described [10], so these correlations are not absolute. As we shall detail below, prion variant differences have been recognized in interspecies or intraspecies transmission, in the response to the overproduction or deficiency of chaperones and other cell components, and in the sensitivity to a growing array of “anti-prion systems”, cell components that cure prions in normal cells, without the overexpression or deficiency of cell components—apparently a complex array of defensive measures against the dangers of prion infection (Table 1).

## 3. Prion Domains, Amyloid Architecture, and Propagation Mechanism

**Figure 1:**
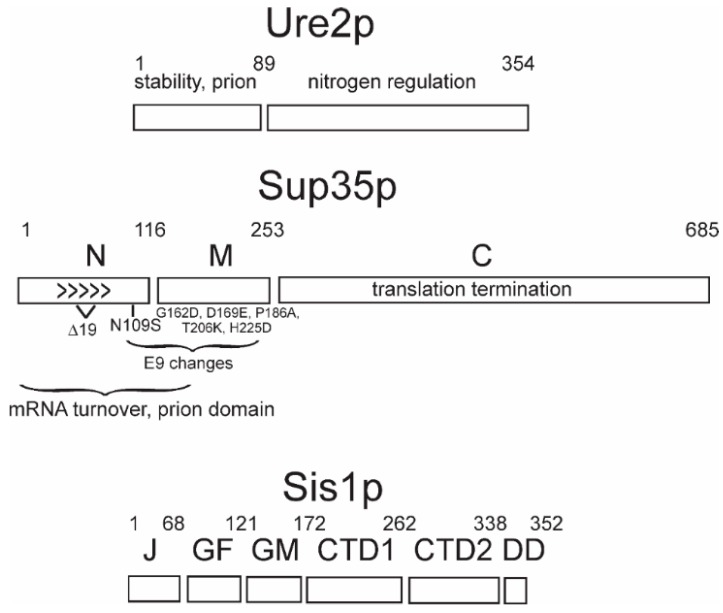
The protein domains: The domains of prion-forming proteins Ure2p and Sup35p and the Hsp40 family member, Sis1p, are shown. For Sup35p, the sequence difference for the natural variants E9 and 19Δ, compared to the reference (lab strain) sequence, are shown.

Each prion protein has a distinct domain that is necessary for the propagation of the prion form, and that generally coincides with the part of the protein that forms amyloids [9,13,15,17,45,46,47] (Figure 1). Amyloid is a linear polymer of a single protein or peptide, with a largely β-sheet structure having the β-strands perpendicular to the long axis of the filaments. Within this definition, there are a variety of possible architectures for amyloids (reviewed in Reference [48]). β-sheets in enzymes are most commonly antiparallel, but this architecture is unknown in natural amyloids. Most pathogenic amyloids are parallel in-register β-sheets [49], including the yeast infectious amyloids of Sup35p, Ure2p, and Rnq1p [50,51,52,53,54,55] (Figure 1). In contrast, the infectious HET-s prion domain amyloid is a two-turn per molecule β-helix [56,57].

**Figure 2:**
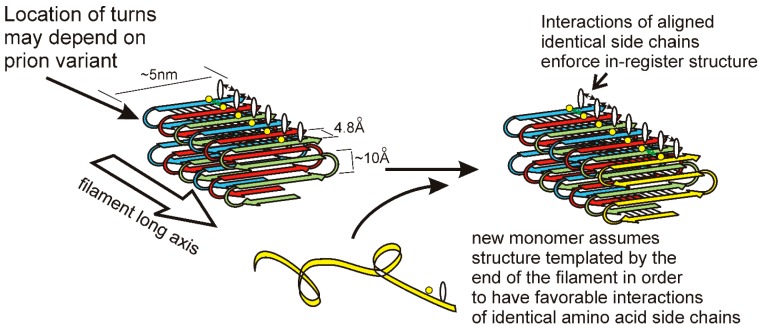
A model for the templating of prion variant information: Yeast prion infectious amyloid has the folded in-register parallel β sheet architecture depicted here. This structure is maintained by the favorable interactions among identical amino acid side chains that requires them to be in-register. If the locations of the folds in the sheet (turns of the peptide chain) determine the prion variant, then the end of the filament will template the folding of a monomer joining the end of the filament by requiring the same favorable interactions of identical sidechains [61]. Reprinted with permission from Wickner, RB et al., *Biochemistry* 52, 1514–1527 (2013). Copyright 2013 American Chemical Society.

The in-register parallel β-sheet architecture features rows of sidechains of identical amino acids along the long axis of the filament (Figure 2). Such rows result in (or actually exist because of) favorable interactions among the identical side chains, including hydrophobic interactions for hydrophobic residues or a line of hydrogen bonds for N, Q, S, or T residues. Only charged residues will have an unfavorable such interaction from charge repulsion, and there are remarkably few charged residues in these prion domains [7,45]. Indeed, point mutations in the Sup35p prion domain that could not propagate a variant of [PSI+] produced largely G to D or Q to E mutations [58,59]. It is the rows of hydrogen bonds and the rows of hydrophobic interactions, extending the length of the filaments, which maintain the register of the in-register parallel structure. We have proposed [60,61] that it is these same interactions that force the unstructured prion domain [62] to have the same turns as the molecules already in the filament. We suggest that different prion variants have the folds of the sheet (turns in the β-strands) in distinct locations along the peptide chain. This constitutes conformational templating and allows prions to act as genes with multiple alleles (multiple amyloid conformations = multiple variants/strains) with different biological properties. There is also clear evidence that different prion variants can differ in the domains that are highly structured [55,63]. The distinct properties of different prion variants may be due to different exposed residues, distinct chaperones and other factors bound to the filaments, and a higher or lower energy of binding monomers.

## 4. Detrimental Prions Have Variants, but Not the Beneficial [Het-s] Prion

The mammalian and yeast prions each have many prion variants, but, as expected of a functional prion, there is only one variant of [Het-s] [64]. The HET-s prion domain forms a unique structure in vitro [56,57], while the prion domains of Sup35p and Ure2p form a mixture of structures (although all seem to be folded in-register parallel β sheets) [50,51]. A functional prion is selected to have a specific structure, one that optimizes its function, but prions that are molecular accidents may have many conformations/variants. The intramolecular bonds of the HET-s β-helix probably form first, fixing the basic architecture, followed by the intermolecular bonds [56]. A knee bends in a very specific way, but a leg may be broken in many different ways.

## 5. Degree of Pathogenicity Varies with Prion Variant

Although it has been proposed that [PSI+] can be beneficial to its host [65,66,67], the experimental basis for these claims, the improved growth of certain [PSI+] strains under certain circumstances, has not been confirmed [68]. The rare occurrence of the [URE3], [PSI+], and [PIN+] prions in wild strains, combined with their spread by non-chromosomal segregation and arising spontaneously at about 1 in 10^6^ cells (precluding geographic isolation) implies that even the mildest variants of these prions are detrimental to their hosts ([69,70]; reviewed in Reference [61]). In contrast, the functional [Het-s] prion is found in 95% of wild strains of the appropriate chromosomal genotype [71], as expected for a functional prion.

In a screen designed to include [PSI+] variants in which too much of the essential Sup35 protein was sequestered in filaments, the majority of isolates were extremely toxic to cells or even lethal [33]. Similarly, most [URE3] variants dramatically slow growth although some only a slight affect cell growth [33]. The fact that the nonessential Ure2p can become a very toxic prion proves that yeast prions, like mammalian prions, are not detrimental solely by the deficiency of the normal protein function. Moreover, it is likely that in these screens, there were many lethal variants which were not detected, but at least the results say that retaining the prion-forming ability entails a substantial cost. The effects of lethal variants have generally not been considered in models asserting that [PSI+] can be beneficial to its hosts.

## 6. Interspecies Transmission Barriers Vary with Prion Variant

Early studies in mammalian prions showed that barriers to the transmission of scrapie between species [72] were due to differences in sequence between the PrP of the donor and recipient [73]. Yeast prions also show species barriers to transmission based on prion protein sequence differences [34,74,75,76,77]. King showed that a panel of mutations in the prion domain of Sup35 could be used as a method to identify or type prion variants [76].

The various species of the genus *Saccharomyces* are known to mate with each other quite efficiently, although the resulting hybrid diploids produce few viable spores. Thus, the spread of prions among these species is likely to occur. [URE3] prions originating from the Ure2p of the various *Saccharomyces* species transmit well to the same Ure2p but poorly or, in some cases, not at all to the Ure2p of another species, but the extent of this effect depends on the prion variant [34]. For example, a [URE3cer]^bay^, namely a [URE3] originating in *S. cerevisiae* but propagating in a cell expressing the Ure2p of *S. bayanus*, transmits well to a cell expressing Ure2p^cerevisiae^. However, [URE3bay]^bay^ will transmit well to another cell expressing Ure2p^S. bayanus^, but not at all to a cell expressing Ure2p^S. cerevisiae^ [34]. Prions also seem to have a “memory” of their sequence of origin. The species barriers also depended on the individual variant even when the species of origin were identical. As would be expected, species barriers were generally asymmetrical [34].

## 7. Intraspecies Transmission Barriers and Prion Variants

Sequences of the *SUP35* gene of 70 wild *Saccharomyces* strains showed that variation of the N and M domains is far more frequent than of the C domain [35]. The C domain is the part of the protein that is essential for translation termination [78]; therefore, part of the explanation for its less frequent change could be the need to conserve this function. However, the N and part of M domains are involved in [PSI+] propagation, and even single amino acid differences in these regions between prion donor and recipient can result in a block of prion propagation [58,59] (Figure 1). There are three broad groups of *SUP35* alleles among the 70 wild strains examined: the reference allele (found in most lab strains), Δ19 (a polymorph of Sup35p having a deletion of residues 59–77 in the Sup35p N), and E9 (N109S and 4 changes in the M domain). The [PSI+] prion can arise in any of these Sup35p sequence polymorphs, but the transmission (infection) of [PSI+] variants generated in a strain with one polymorph into a strain carrying another polymorph is generally inefficient [35]. This “intraspecies transmission barrier” depends very much on the prion variant. For example, a [PSI+Δ19]^Δ19^ (isolated in cells with the Δ19 polymorph of Sup35p and propagated in cells expressing this polymorph) transmits very poorly to either of the other two natural polymorphs of Sup35p, but a [PSI+ref]^Δ19^ (originating in a strain with the Sup35p sequence of lab strains (reference) but propagating in the Sup35p polymorph with the deletion) transmits very well to the other polymorphs [35]. The decimation of the Fore population by Kuru, the spongiform encephalopathy transmitted by funeral feasts, has resulted in the selection of a mutation of PrP residue 127 conferring resistance to the disease [79]. Likewise, it is possible that the sequence polymorphisms in the N and M domains have been selected to protect from infection with [PSI+] [35]. In fact, some yeast and fungal species have Sup35 and Ure2 proteins that cannot form prions at all [34,80]. More details of the basis for considering [PSI+] and [URE3] as diseases of yeast may be found in Reference [61].

## 8. Prion Mutation and Segregation of Variants

A given prion strain/variant generally propagates quite stably, maintaining its properties over time. However, it has long been known that under conditions of selective pressure, such as the introduction into a largely incompatible host (species barrier), prions can mutate, that is, change their properties, in a heritable manner. For example, two generations of passage in hamsters of a mouse-adapted strain of scrapie resulted in an altered prion strain on its return to mice, presumably because of the selection pressure of replicating in hamsters [81]. Likewise, treatment with the amyloid-binding drug swansonine seems to select drug-resistant scrapie prion strains from a strain that was drug-sensitive before exposure [82]. Yeast prions passed to a cell expressing a prion protein with a different sequence (species barrier or intraspecies barrier) can lead to the selection/development of prions no longer restricted on the new host (e.g., in References [34,35]). However, in all these, cases it is difficult to distinguish the effect of the assay (drug treatment and propagation with the new prion protein sequence) from the mutational event. It is possible that the assay is producing the mutant rather than merely detecting and selecting it.

**Figure 3:**
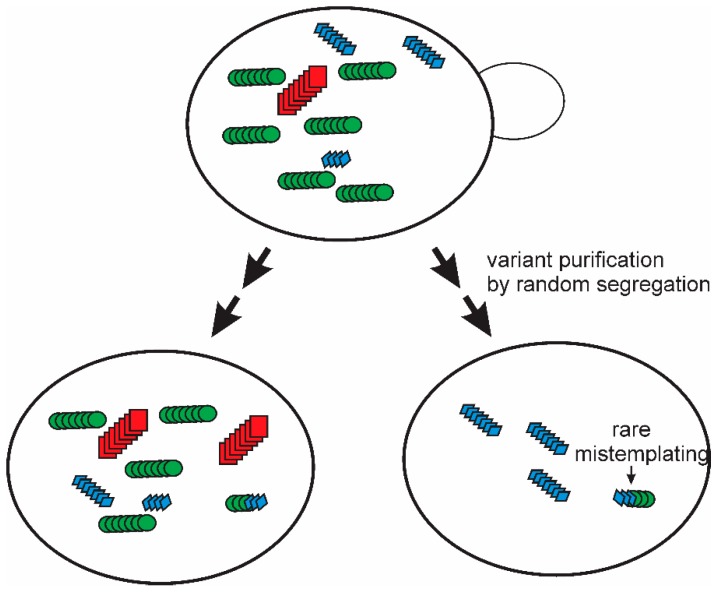
The prion cloud model: The segregation and mutation observed for [PSI+] variants examined for their sensitivity to intraspecies barriers based on a variation of the sequence of Sup35p [36] provided strong evidence for the “prion cloud” model proposed by Collinge for mammalian prions to explain species barriers [83,84]. The same model explains many of the prion mutation/segregation phenomena reviewed here. Modified from Reference [36].

A single multiply cloned [PSI+] prion variant isolated using the “reference” lab strain Sup35p, showed a limited transmission to other polymorhps of Sup35p [35]. It was found that simple subcloning and extensive propagation of that [PSI+ref]^ref^ strain led to the appearance of mitotic segregants of the four logical types: A) a poor transmission to either other polymorph, B) a good transmission to the Δ19 polymorph but poor transmission to the E9 polymorph, C) a poor transmission to the Δ19 polymorph but good transmission to E9, and D) a relatively good transmission to both Δ19 and E9 [36]. But these types were not completely stable. Further mitotic growth of each of these strains resulted in the generation, again, of all four types from each one. In these experiments, the test of transmission was clearly separated from the process of prion change-of-variant. This means that there can be no question of the test altering the prion. Thus, these experiments imply that the “prion cloud” model [83,84] is true at least for yeast prions (Figure 3). Even an apparently pure prion variant/strain is a mixture, and purification of one variant does not last, as mutation evidently occurs on further propagation [36].

Prion mutation is seen in other contexts as well. The toxic [PSI+] and [URE3] variants gradually become more mild [33]; [URE3] variants sensitive to normal levels of Btn2p and Cur1p (see below) frequently lose that sensitivity, even in a *btn2*Δ *cur1*Δ double mutant [42]; and [PSI+] variants cured by normal levels of the disaggregase Hsp104 become insensitive, even when propagated in a strain where the Hsp104 curing activity is disabled [41] (see below). Sharma and Liebman have described a [PSI+] variant that, during growth, continues indefinitely to segregate both strong and weak variants [85].

## 9. Prion Variant Generation

In yeast, the generation of prions can be roughly synchronized because an overproduction of the prion protein generally induces the appearance of the respective prion at dramatically increased frequencies [3]. The overexpression of Sup35p or its prion domain produces ring-shaped aggregates in a portion of cells, and these cells generally are or become [PSI+] [86,87]. By following the pedigrees of such cells, approx. 40% were found to give rise to both strong and weak variants among their progeny, suggesting multiple prion generation events in a single cell followed by segregation [85]. If weak and strong variants from separate cells are combined in a single cell, the strong variant generally prevails, probably because its more-ready fragmentation generates more ends, which, in turn, succeeds in competition for monomers [28].

## 10. Effects of Chaperones and Other Proteins on Prions

Hsp104 is a disaggregating chaperone which, together with Hsp70s and Hsp40s, extracts monomers from an aggregate, giving them a chance to refold properly [88,89,90,91]. In extracting a monomer from the middle of a prion filament, Hsp104 splits the filament into two, a process necessary for the propagation of all of the yeast amyloid-based prions [92,93,94]. The cytoplasmic Hsp70s (Ssa’s) [95,96,97,98,99] and the Hsp40s Sis1p, Ydj1p, and Swa2p [98,100,101] are also needed for the propagation of yeast prions (Table 2). The yeast Hsp104 and Hsp70s and their nucleotide exchange factors work together in supporting prion propagation as shown by the ability of their *E. coli* homologs (ClpB, DnaK, and GrpE) to substitute as a group but not individually in this activity [102]. Hsp90 and its cochaperones Cpr7p and Swa2p are needed for [URE3] propagation but not for [PSI+] [101,103].

The overproduction of Hsp104 also cures the [PSI+] prion [104] (and [URE3], inefficiently [110]). The deletion of or mutations (e.g., *hsp104(T160M))* in the N-terminal domain of Hsp104 disable the overproduction curing of [PSI+] but not the ability to propagate this prion [111], thus showing that these are two distinct activities. The mechanism of the Hsp104 overproduction curing of [PSI+] is controversial [99,112,113,114] but may involve the asymmetric segregation of prion filaments and/or the interference with Hsp70 chaperone binding in the filament fission reaction. The Hsp104 overproduction curing of [PSI+] (but not its role in prion propagation) requires Apj1p (an Hsp40) [105] and the action of Hsp90 and its cochaperone, Sti1p, but the precise role of these proteins in the process are not yet clear [108,115]. Apj1p is needed for the Hsp104 overproduction-curing of strong [PSI+] variants but not for weak variants [80], and Apj1p overproduction cures some [PSI+] variants [116]. Sgt2p, a regulator of the GET pathway, affects Hsp104 overproduction-curing and is induced several-fold by the introduction of the [PSI+] and [PIN+] prions [109].

The overproduction of Ydj1p [117], ribosomal stalk protein Rrp0 and ribosome-associated chaperones [116,118], Sse1p [39], the HOOK-related proteins Btn2p or Cur1p [110], or Hsp42 [42] can result in the loss of prions. In addition, prions may require Cpr7 [103], Sse1p, or Fes1p [39]. In addition, the overproduction of Gpg1p, the gamma subunit of a heterotrimeric G protein [119], cures [PSI+], [URE3], and [PIN+] [120]. The mechanism of this effect is not yet clear, but the overexpression of Hsp104 counters the effect of overexpressing Gpg1p, and other subunits of the G protein do not seem to be involved [120].

## 11. Anti-Prion Systems Normally Block Almost All Prion Variants from Appearing

Although there are numerous proteins whose overproduction or deficiency cures yeast prions, the curing of prions at normal levels of cell components are of particular interest since these represent cellular defenses against yeast prion diseases (“anti-prion systems”; reviewed in Reference [121]). Many of these systems are prion variant-specific.

### 11.1. Ssb Ribosome-Associated Hsp70s Block Prion Formation

In the absence of Ssb1p and Ssb2p, there is an accumulation of aggregated proteins in otherwise unstressed cells [122]. The Ssb’s are believed to assist the proper folding of nascent polypeptides, and in their absence, the frequency of [PSI+] arising de novo is increased ten-fold [123]. The replacement of normal levels of Ssb1p does not cure any of the prions that arose in its absence [123], although [PSI+] can be cured by the overproduction of Ssb1p [124]. Thus, Ssb’s block the generation of the [PSI+] prion in a variant nonspecific way.

### 11.2. Normal Levels of Btn2p and Cur1p Cure the [URE3] Variants with Low Seed Number

The overproduction of Btn2p or Cur1p, paralogs with a distant relation to the human HOOK1 protein, can efficiently cure any known variant of [URE3] [110]. Btn2p acts by collecting the Ure2p amyloid filaments in one place in the cell so that, on cell division, one of the daughter cells often receives no amyloid and, so, is cured [110]. Btn2p also collects non-amyloid aggregates of optineurin (related to amyotrophic lateral sclerosis), with Btn2p reducing their toxicity, as well as aggregates of PrP and polyQ [125]. Btn2p was previously found to be involved in protein trafficking between late endosomes and the Golgi [126], but the relation of this activity to its aggregate-collecting activity is as yet unclear.

In the absence of Btn2p and Cur1p, the [URE3] prion arises approx. 5 times more frequently than in their presence, and about 90% of such [URE3]s are cured by merely restoring the normal amount of both proteins [42]. Those [URE3] variants that are cured in a normal cell by Btn2p are all weak in phenotype and have a low seed number [42], in support of the amyloid sequestration model of Btn2p curing [110]. It is inferred that [URE3] prions are very frequently arising de novo but that these anti-prion systems are constantly culling all but those with the highest seed number.

The propagation of Btn2/Cur1 hypersensitive [URE3] variants in a *btn2*Δ *cur1*Δ strain, presumably a permissive condition, results in the appearance of several altered variants, including those which are no longer sensitive to Btn2 or Cur1 for propagation [42]. Like the studies of changes in the susceptibility of [PSI+] variants to intraspecies barriers and to Hsp104 curing, this reflects the mutation and segregation of prion variants during growth.

### 11.3. Normal Levels of Hsp104 Cull Many [PSI+] Prion Variants

Recently, it has been shown that, without its overproduction, normal levels of Hsp104 eliminate as much as 90% of [PSI+] variants arising spontaneously by a mechanism that resembles the overproduction curing in its requirements for Hsp90 and its cochaperone, Sti1p [41]. [PSI+] variants were generated in an hsp104(T160M) mutant [111], inactivated for the overproduction curing activity. About half of the [PSI+] variants were lost on transfer to a wild type host, and the frequency of [PSI+] arising spontaneously was >10-fold higher in hsp104(T160M) mutants than in wild-type cells [41]. The variants of [PSI+] eliminated by this Hsp104 activity include both “strong” and “weak” [PSI+], and, unlike the curing of [URE3] by normal levels of Btn2p and Cur1p, there is no correlation with seed number. [PSI+] variants curable by normal levels of Hsp104 are unstable, even in the hsp104 T160M mutant, and gradually become insensitive to the curing [41].

### 11.4. Normal Levels of Upf Proteins Cure Most Spontaneous Variants of [PSI+]

A general screen was carried out for anti-prion components that, in a normal cell, cure [PSI+] variants that arise in cells with a knockout mutation. Upf1 and Upf3, components of the nonsense-mediated mRNA decay system, were frequently detected, and *upf2* mutants had the same property [43]. [PSI+] arose with a >10-fold higher frequency in *upf*Δ strains than in wild-type cells, and over 90% of those arising were cured by restoring the normal level of the Upf protein. The inability to cure these Upf-hypersensitive variants did not correlate with a lack of nonsense-mediated decay but did correlate with the failure to form the Upf1,2,3-Sup35 complex that is involved in the process [43]. The lesson from these results is that normal protein–protein interactions can prevent, or even reverse, the abnormal interactions that are involved in prion/amyloid formation.

### 11.5. Inositol Poly/Pyro-Phosphates Involvement in [PSI+] Prion Propagation

**Figure 4:**
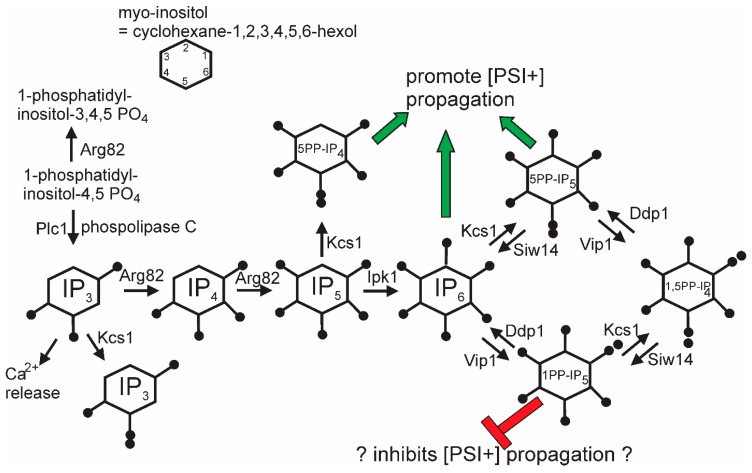
Pathways of inositol poly/pyrophosphate biosynthesis: The green arrows show chemical species that can support [PSI+] propagation, while the red symbols show species that block [PSI+] propagation when 5-pyrophosphates are not made (*kcs1* mutants). Modified from Reference [44].

The general screen for anti-[PSI+] systems that identified the Upf genes also detected *siw14*Δ as defective in curing some [PSI+] variants [44]. *SIW14* encodes a pyrophosphatase specific for 5-diphosphoinositol pentakisphosphate (5PP-IP5, Figure 4), one of the soluble inositol polyphosphate signaling molecules [127]. About half of [PSI+] variants arising in a *siw14*Δ need the elevated level of 5PP-IP5 to propagate, and nearly all variants need some inositol polyphosphates [44]. This requirement is met by either IP6, 5PP-IP4, or 5PP-IP5. In the absence of the 5PP-modified IPs, 1PP-IP6 inhibits [PSI+] propagation. The mechanism of action of the inositol polyphosphates on [PSI+] propagation is as yet unclear, but it is clear that the degree of the requirement is prion variant-dependent.

## 12. Differential Effects of Chaperones on Prion Variants

Sis1p, an essential Hsp40 in yeast, is required for the propagation of [PSI+], [URE3], and [PIN+] [100] and is part of the Hsp104-Hsp70-Hsp40 apparatus that splits filaments making new growing points. The Sis1 J and GF or J and GM domains (Figure 1) are sufficient for cell growth, and various combinations of domains have been examined for their effects on yeast prions [37,38,128,129]. The deletion of GM and the C-terminal domain results in the loss of a weak [PSI+] variant, while a strong [PSI+] is not lost but is lethal. In contrast, *sis1*ΔGF maintains the [PSI+] variants but loses all [PIN+] variants. The deletion of GM or of CTD loses some [PIN+] variants and retains others. Thus, Sis1p protects cells from the potential lethality of strong [PSI+], and Sis1p mutations produce a differential loss of variants of [PSI+] or [PIN+].

In *sse1*Δ, a weak [PSI+] variant was lost but a strong variant was weakened but not lost [39]. In other studies, Sse1p overproduction stimulated [PSI+] generation, and its deficiency resulted in only certain weak variants arising [40,130].

## 13. The Chaperone Environment Selects Prion Variants

Chaperones are known to have an array of strong influences on prions, with the overexpression or deficiency of certain chaperones curing or inducing the appearance of various prions (Table 2). The [PIN+] prion of Rnq1p is detected by its ability to cross-seed the formation of the [PSI+] prion on the overexpression of Sup35p [23]. [PIN+] has variants with different efficiencies of priming [PSI+] formation, called “high”, “medium”, and “low” [30]. Interestingly, deletions of certain chaperones can result in the change of one [PIN+] variant to another [106]. The deletion of *HSC82* (the constitutive Hsp90), *AHA1*, *CPR6*, *CPR7* (cochaperones of Hsp90), or *TAH1* (component of the R2TP complex that interacts with Hsp90) all result in the change of a low or medium variant to a high variant. In contrast, the deletion of another Hsp90 cochaperone gene, *SBA1*, changes a high variant to a low variant [106]. These results correlate with the known stimulation of Hsp90’s ATPase by Aha1p, Cpr6p, and Cpr7p and its inhibition by Sba1p (see Reference [106]). The deletion of *SSE1* (encoding the Hsp70—a related nucleotide exchange factor p110) also converted a low variant to a high variant [106].

It was not just the [PIN+] variant phenotype that was changed by these deletions, as the transfer of the prion to a wild type strain maintained the new variant traits [106]. Two-hybrid interactions of Rnq1p with Sba1p, Tah1p, and Cpr7p were also observed. This work supports a prominent role for Hsp90 in prion propagation, as suggested first in studies of the Hsp104 overproduction curing of [PSI+] [108,115] and, later, in the requirement of [URE3] for the Hsp90 cochaperone Cpr7 [103]. It also shows that prions can mutate, similar to conclusions reached in a contemporaneous study on [PSI+] and intraspecies barriers [36].

## 14. Prions Are More Abundant and Varied than Was Previously Thought

**Figure 5:**
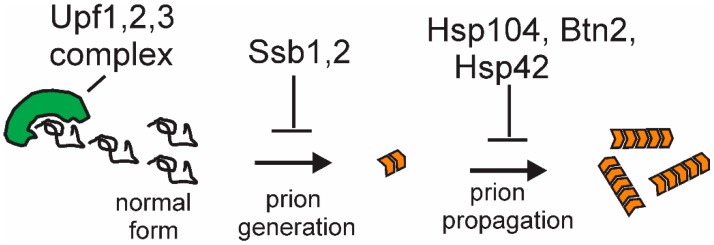
The mechanisms of anti-prion system action: Although detailed mechanisms are as yet unclear, the Upf proteins appear to compete with filaments for Sup35p monomers or block the ends of growing filaments; Ssb1,2, in facilitating the folding of nascent proteins, prevent Sup35p misfolding; and Btn2p (with Hsp42) collects aggregates at one cellular locus. The mechanism of Hsp104’s antiprion action is discussed in the text.

Recent studies of anti-prion systems (cellular systems curing prions in normal cells) indicate that there are multiple systems working at different stages in the prion generation/propagation process (Figure 5) and that the number of prions arising is far greater than had been previously suspected. The *hsp104(T160M)* mutants produce [PSI+] at >10-fold the normal spontaneous rate [41], *upf* mutants at approx. 15-fold the normal rate [43], and *siw14* mutants at, at least, twice the normal rate [44], in each case producing mostly prions that are cured by replacing the normal level of the normal protein. Are these all the same new variants? It is possible that there is overlap but likely that any overlap is only partial. The [PSI+uss] (Upf-super-sensitive) variants arise in cells with normal Hsp104 and normal inositol polyphosphate genes and similarly for the other cases. Thus, there appear to be an abundance of [PSI+] and [URE3] variants arising, with the continuing elimination of most new prions and only a few persisting. This situation is quite similar to the array of DNA repair systems correcting most lesions, with only a few persisting, each DNA repair system specific for a particular kind of lesion. It will be important to understand the detailed structure of prion variants beyond the general patterns now seen and to elucidate the detailed mechanisms of the various anti-prion systems.

## 15. Implications for Human Disease

There are no prion-curing systems yet known in humans, but there is a vast array of chaperones (most closely homologous to those of yeast), inositol polyphosphate pathways nearly identical to those in yeast, and proteins that normally associate with prion or amyloid-forming proteins (as the Upf proteins associate with Sup35p). It is likely that prions and amyloids are constantly arising and being eliminated until either a specific variant arises that is resistant to anti-prion systems or an anti-prion system becomes defective due to aging or disease. The situation is largely parallel to the various innate and adaptive immune systems and their complex interaction with viruses, bacteria, and other infectious agents. The one difference is that the infectious agent in the case of the prions and amyloids is an endogenous protein rather than an outside invader.

## Figures and Tables

**Table 1:** Yeast prion variant classification.

Variant-Defining Condition/Trait	Prions Affected	Mechanism	Relation to Strong/Weak?	Refs.
Strength of phenotype (strong/weak)	[PSI+], [URE3]	high filament number adsorb more prion protein		[10,28,29]
Prion stability	[PSI+], [URE3]	high filament number insures both daughters infected	Strong often stable, weak often unstable; exceptions	[10,28,29]
prion toxicity (all variants detrimental but the degree varies)	[PSI+], [URE3]	[PSI+]: depletion of Sup35p (essential) [URE3]: toxic effect of amyloid form	unknown	[33]
interspecies or intraspecies barriers	[PSI+], [URE3]	inefficient binding to amyloid of different protein sequence		[34,35,36]
lethality or prion loss in Sis1p partial deletions	[PSI+]	unknown	strong variant lethal; weak variant lost	[37,38]
Sse1p required for propagation or generation	[PSI+]		strong variant weakened, weak variant lost	[39,40]
curing by normal levels of Hsp104	[PSI+]		some of both are cured; no relation to seed #	[41]
curing by normal levels of Btn2p	[URE3]	filaments sequestered by Btn1p, Hsp42p	All cured variants are weak, but some weak variants not cured	[42]
curing by normal levels of Cur1p	[URE3]	unknown	All cured variants are weak, but some weak variants not cured	[42]
curing by normal levels of Upf1,2,3	[PSI+]	complex formation with Sup35p	no relation to strong/weak	[43]
curing by normal levels of Siw14p	[PSI+]	limits 5PP-IP5 levels	unknown	[44]

**Table 2:** The effects of Chaperones on yeast prions.

Chaperone	Effects	Prions Affected	Refs
Hsp104	filament cleavage (with Hsp70 and Hsp40)	all amyloid-based yeast prions	[104]
Ssa1–4 (Hsp70)	filament cleavage	[PSI+] and [URE3]	[96]
Sis1 (Hsp40)	propagation; needed for Hsp104 curing; prevents toxicity	all amyloid-based yeast prions; [PSI+]; strong [PSI+]	[37,100]
Swa2 (Hsp40)	propagation	[URE3]	[101]
Apj1 (Hsp40)	needed for Hsp104 curing of strong [PSI+]	[PSI+]	[105]
Hsp90	needed for Hsp104 curing; variant selection; propagation	[PSI+], [PIN+], and [URE3]	[101,103,106,107]
Sti1	needed for Hsp104 curing	[PSI+]	[108]
Cpr7	Hsp90 co-chaperone	[URE3]	[103]
Fes1	overproduction curing	[URE3]	[39]
Sse1	propagation: necessary and overproduction curing	[URE3] and [PSI+]	[39,40]
Sgt2	affects Hsp104 curing; induced by prions	[PSI+] and [PIN+]	[109]
